# Computational Fluid Dynamics Analysis of Alternative Ventilation Schemes in Cage-Free Poultry Housing

**DOI:** 10.3390/ani11082352

**Published:** 2021-08-09

**Authors:** Long Chen, Eileen E. Fabian-Wheeler, John M. Cimbala, Dan Hofstetter, Paul Patterson

**Affiliations:** 1 Department of Agricultural and Biological Engineering, The Pennsylvania State University, State College, PA 16802, USA; dwh5212@psu.edu; 2 Tianjin Academy of Agricultural Sciences, Tianjin 300384, China; 3 Department of Mechanical Engineering, The Pennsylvania State University, State College, PA 16802, USA; jmc6@psu.edu; 4 Department of Animal Science, The Pennsylvania State University, State College, PA 16802, USA; php1@psu.edu

**Keywords:** ventilation system, cage-free hen housing, computational fluid dynamics, full-scale simulation, indoor air conditions, bird welfare

## Abstract

**Simple Summary:**

Uncertainty regarding “cage-free” housing guidelines have left egg producers unsure about how to transition to cage-free housing. A primary driving force behind cage-free housing is the perceived animal welfare concerns for caged birds. Therefore, it is of great importance to perform a systematic investigation on specific cage-free facility types with an emphasis on bird comfort assessment. Thus, the goal of this study was to investigate alternative ventilation schemes of a cage-free house to provide practical designs for a comfortable interior environment at the hen level. By modeling four different ventilation schemes in a one-eighth section of a typical floor-raised layer house—indoor temperature, air speed, and static pressure were compared and analyzed quantitatively. Distribution contours and quantitative analysis of airflow, temperature, and pressure suggested that indoor conditions could be maintained at a suitable range uniformly, especially at the hen level. In addition, the ventilation rates of the hen house within four ventilation schemes fell at the higher end of the desired ventilation range, indicating that the barn could be expected to maintain good air quality during cold weather. This study demonstrated that computational fluid dynamics modeling was a powerful tool that facilitated researchers to address animal welfare issues in animal housing designs.

**Abstract:**

This work investigated alternative ventilation schemes to help define a proper ventilation system design in cage-free hen houses with the goal of assuring bird welfare through comfortable conditions. Computational fluid dynamics (CFD) modeling was employed to simulate indoor and outdoor airflows to quantify the effectiveness of ventilation systems in maintaining suitable and uniform living conditions at the hen level. Four three-dimensional CFD models were developed based on a full-scale floor-raised layer house, corresponding to ventilation schemes of the standard top-wall inlet, sidewall exhaust, and three alternatives: mid-wall inlet, ceiling exhaust; mid-wall inlet, ridge exhaust; and mid-wall inlet, attic exhaust with potential for pre-treatment of exhaust air. In a sophisticated and powerful achievement of the analysis, 2365 birds were individually modeled with simplified bird-shapes to represent a realistic number, body heat, and airflow obstruction of hens housed. The simulated ventilation rate for the layer house models was 1.9–2.0 m^3^/s (4100 ft^3^/min) in the desired range for cold weather (0 °C). Simulation results and subsequent analyses demonstrated that these alternative models had the capacity to create satisfactory comfortable temperature and air velocity at the hen level. A full-scale CFD model with individual hen models presented robustness in evaluating bird welfare conditions.

## 1. Introduction

Poultry facilities are going through significant transitions to cage-free production modes to address bird welfare concerns with caged housing. Aviary systems, convertible cages, and floor housing systems are three representative cage-free housing systems that are commonly used [1]. Apparently, each configuration has its merits with regard to stocking densities, mortality rates, and disease control, yet with nuisances, such as eggs laid outside of nest-boxes (a.k.a. floor eggs). The lack of unified guidelines and industry conflict regarding what “cage-free” means have left egg producers without clear understanding about how to proceed the transition to cage-free housing, or to which particular system to switch. Furthermore, both egg producers and poultry house contractors are confronting difficulties in finding the most effective designs of cage-free facilities to maintain optimal production along with excellent bird comfort and welfare [2]. Thereby, current ventilation systems have not necessarily kept pace with the new poultry buildings in this era due to a lack of performance-based systematic design procedures.

Environmental control ventilation systems are vital in cage-free poultry production [1]. A bird’s homeothermic condition is highly determined by air temperature, relative humidity, thermal radiation, and air movement inside the poultry house. Hens are homeothermic animals, which are susceptible to heat or cold stress that cause physiological disorders. In fact, any key environmental factor that is off its optimal range may undermine bird welfare and production performance [3]. In addition to maintaining a desired thermal condition, uniform indoor environmental conditions in poultry housing are crucial, particularly considering the increasing size of commercial poultry barns [4]. Therefore, the design of a ventilation system is of paramount importance since the characteristics and uniformity of environmental parameters are driven by indoor airflow patterns [5]. In fact, fluctuating production rate is commonly found with cage-free housing due to unevenly distributed indoor environmental influences, such as uneven free-roaming hen density.

Computational fluid dynamics (CFD) has been used as a powerful tool to model fluid flow in diverse applications. For decades, CFD modeling has been employed to address indoor environmental problems and optimize the design of ventilation systems in a variety of agricultural facilities [6]. Most environment influencing factors are controllable in the CFD model and universal outputs of interest can be obtained. Moreover, CFD modeling is relatively low-cost in terms of time and computational expenses compared to testing constructed facilities. Numerous studies have demonstrated that CFD models can reliably predict airflow, heat, and mass transfer in animal housing systems [7,8,9,10].

The need to address indoor environment problems in poultry housing has encouraged researchers to improve the design of ventilation systems by CFD modeling. Research conducted by Mistriotis and Jong investigated a broiler house with natural ventilation by developing a two-dimensional CFD model, and the simulation results revealed that the uniformity of indoor temperature and air velocity distribution was improved by installing a solar chimney [6]. Blanes-Vidal, Guijarro, Balasch, and Torres (2008) conducted four CFD simulations of airflows in a mechanically ventilated commercial poultry house to evaluate whether air velocities in European poultry houses fell within the optimal zone [11]. Seo et al., (2009) modeled four modified ventilation systems for a naturally ventilated broiler house, and they found that the model with a diffuser beneath the chimney inlet had the optimum performance [12]. CFD models have also been used to optimize ventilation systems for extreme weather conditions to address heat stress and cold stress issues [13,14]. All of these studies have demonstrated that CFD models were capable of investigating ventilation options for a variety of poultry houses. However, one of the most challenging hurdles is the bird. Limited studies have evaluated housing performance with an emphasis on bird welfare at a full-scale, since modeling individual birds requires significant computational resources. Additionally, to accommodate the shift to cage-free production, improved and dedicated ventilation designs are in great demand from a bird welfare perspective.

This study applied CFD simulations in characterizing three alternative ventilation schemes applied to a floor-raised layer house, in comparison with the standard ventilation scheme [1] by evaluating indoor environmental conditions within each design. Simulation results of critical environmental parameters for bird comfort assessment, including air speed and temperature, along with the driving force for ventilation air exchange (static pressure difference), were analyzed visually and quantitatively at the whole house and hen levels. The goal was to assess the performance of these ventilation options for cage-free poultry houses to maintain a desired and comfortable indoor microclimate that satisfies the needs of production demand and ensures good bird welfare. Conditions during cold weather were modeled since it was the most challenging period for maintaining comfortable, healthy indoor conditions for hens, due to low ventilation air exchange and fresh air distribution inside the hen house.

## 2. Materials and Methods

Four CFD models and corresponding simulations were conducted using the commercial software package FLUENT v19.1 [15]. The standard k−ε turbulence model [16,17] with enhanced wall functions was adopted for the development of CFD models, based on previous investigations [18,19,20,21]. Simulations were conducted on the Pennsylvania State University’s Institute for Computational and Data Sciences’ Roar supercomputer.

### 2.1. Development of CFD Model

#### 2.1.1. The Floor-Raised Layer House

The modeled layer house was located at Lititz, Pennsylvania, with a typical floor-raised housing configuration [1,22]. The length and width of the layer house were 162.15 m (532 ft) and 13.72 m (45 ft). The sidewall was 2.73 m (8.96 ft) tall, and its thickness was 0.19 m (7.5 in.). In addition, the layer house had a flat interior ceiling and shallow 4/12 exterior roof slope. Typically, almost 20,000 hens were housed in the barn with a stocking density of 0.11 m^2^/bird (1.20 ft^2^/bird).

The current ventilation system of the layer house included 84 rectangular ventilation inlets [each 1.17 m (46 in.) by 0.20 m (8 in.)] located at the top of each sidewall near the eaves along both sides of the building. Four exhaust fans (0.91 m (36 in.) in diameter) installed along one long sidewall of the barn were operated during brooding, and for cold and mild weather. In addition, a tunnel ventilation system was equipped (not modeled in this study) for warmer and hot weather. Fresh outdoor air was drawn into this negative pressure house through the inlets under the barn eaves with sidewall exhaust fans in the standard ventilation scheme used in North America that we referred to as “top-wall inlet sidewall exhaust” [TISE] [1].

#### 2.1.2. Establish the Computational Domain

Initially, a two-dimensional computational domain was employed to simulate the indoor air conditions within TISE ventilation scheme, though the real ventilation performance of the layer house could not be accurately reflected [22]. Thereby, subsequent three-dimensional models of the study barn with standard and alternative ventilation schemes were developed to approach more accurate and realistic simulations [1,22].

A three-dimensional geometry was developed with realistic dimensions provided by collaborators to represent one-eighth of the entire layer house, resulting in a reasonable model size with ventilation features that included an exhaust fan and the proportional quantity of inlets. Only a central section of the layer house was modeled to minimize end-wall effects. The house was modeled using dimensions obtained from construction blueprints. Details of the barn dimensions were described in our previous publication [1].

**Figure 1:**
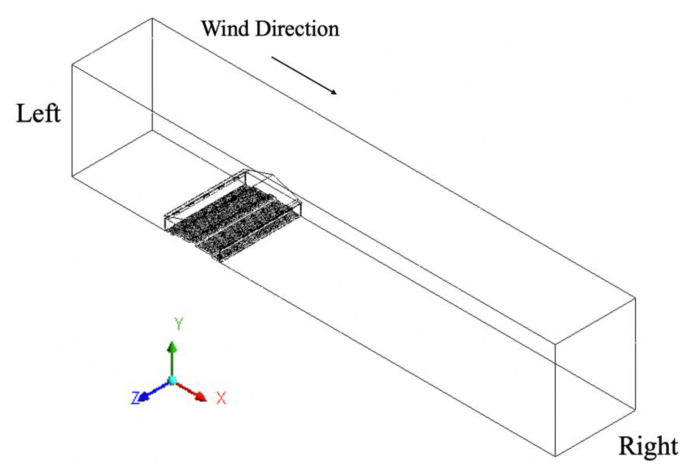
The modeled layer house within the computational domain (TISE).

The computational domain of each model included the barn itself and ambient air to properly simulate airflows inside and outside the building (Figure 1). All models shared the same size computational domain where the height was 24.4 m (80 ft) and the width 128.2 m (420.6 ft). A constant wind speed of 2.0 m/s (4.5 mph) was assigned perpendicular to the barn sidewalls, blowing from left to right across the computational domain. Note that an extended domain far from the target layer house at the downwind side was designed to minimize reverse flows at domain boundaries [23].

#### 2.1.3. Design of Ventilation Schemes

**Figure 2:**
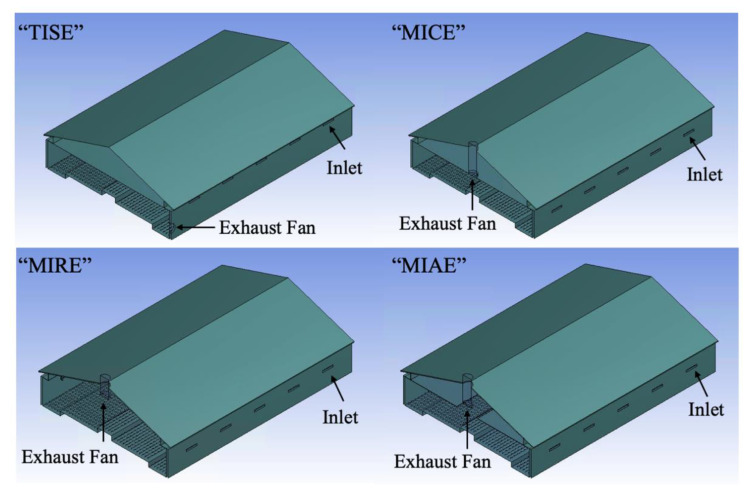
Geometry of the study layer house with the standard ventilation scheme (TISE) and three alternative ventilation designs (MICE, MIRE, and MIAE). Arrows indicate locations of inlets and the exhaust fan.

Four ventilation schemes were modeled and investigated (Figure 2). Other than the standard TISE model [1], three alternative ventilation systems were designed, including “mid-wall inlet ceiling exhaust” [MICE], “mid-wall inlet ridge exhaust” [MIRE], and “mid-wall inlet attic exhaust” [MIAE] (Figure 2) [24]. Inlets of MICE were positioned with the base 1.5 m (60 in.) above the floor and had a wall-plate at the top (Figure 2) to direct incoming air horizontally from the inlet opening baffle. The exhaust fan of MICE was positioned at the middle of the ceiling cross-section. Note that the fan chute was modeled as an attached duct whose length was 2.9 m (112.5 in.). Inlets of MIRE and MIAE were modeled identically with those of MICE. However, the major differences between three alternative designs were the positions of exhaust fans. The MICE configuration resembled some ventilation designs more common in European construction, while no ceiling was included in MIRE and its exhaust fan was placed at the middle of the roof, 3.3 m (131 in.) above the nest-boxes, with a short duct length of 1.6 m (64.5 in.). MIAE had a partial ceiling, forming an “attic space” with a 2.3 m (90 in.) opening along the central length of the layer house. The exhaust fan of MIAE with an attached duct length of 2.5 m (100 in.) was positioned at the middle of the roof ridge at a distance of 2.4 m (95.5 in.) above the nest-boxes.

#### 2.1.4. Modeling Individual Hens

**Figure 3:**
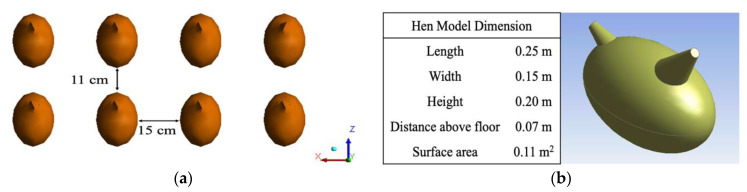
(**a**) Top view of eight evenly distributed hen models; (**b**) dimensions of a single hen model with simplified geometry from isometric view [1]. Reprinted with permission from ref. [1]. 2020. Chen et al.

A significant aspect of the study was to determine conditions at the hen level for welfare comfort conditions, in addition to overall environment patterns in the building air space. For this reason, hen models were included in the simulation. In total 2365 individual hen models were included in each model to represent approximately 1/8 of the total hens housed in the 1/8 house section. Assuming all hens were evenly distributed [1,22], an estimated distance was calculated based on the stocking density shown in Figure 3a. Each hen with an approximate body weight of 1.6 kg (3.5 lb.) was modeled as a heated, simplified hen torso-head-tail-shape [25] with dimensions in Figure 3b. The surface of each hen model was defined with a constant hen body temperature of 42 °C (107.6 °F) and a heat generation rate of 4467 W/m^3^ [23].

#### 2.1.5. Boundary Conditions

For this study, the temperature of the atmosphere was specified as 0 °C (32 °F). Six types of boundary conditions or cell zones were adopted in the CFD simulation inside and outside the layer house [1,22]:

- “Walls”: the ground, ceiling, roof, slatted floor, nesting area, litter area, inlet baffles, sidewalls, animal surfaces, and the top surface of the computational domain [1]. Note that all “walls” were defined as non-slip walls except for the top surface of the computational domain, which was defined as a zero-shear stress wall with no resistance along the surface. One precondition was made by assuming all the walls and roof were ideally insulated. The surface of each hen model was defined with a constant hen body temperature of 42 °C (107.6 °F) and a heat generation rate of 4467 W/m^3^ [23].
- The front and back surfaces of the computational domain along the *z*-axis and both near and far ends of the house were defined as “symmetry” boundary conditions, whereas those surfaces represented internal faces that accounted for 1/8 of the actual scenario.
- Two faces of each inlet that were perpendicular to the wind direction and two faces (functioning parts) of the exhaust fan were assigned boundary conditions of “interior” to represent an interior portion of the computational domain through which air could flow [1].
- “Velocity inlet” was assigned to the left end of the entire domain with a specified wind magnitude of 2.0 m/s (393.7 ft/min) along the positive x-axis (Figure 1).
- “Pressure outlet” was assigned to the right end of the entire domain representing where the flow exits to atmospheric pressure (0 Pa).
- A “3D fan zone” was assigned to the body of exhaust fan where the entire fan volume was considered a fluid cell zone, which simulated the effect of an axial fan by applying a distributed momentum source [15]. Constant pressure jump values of 18 Pa (0.072 in. of water), 20 Pa (0.080 in. of water), 16 Pa (0.064 in. of water), and 15 Pa (0.060 in. of water) were applied across all the cells in the fan zone of TISE, MICE, MIRE, and MIAE models, respectively [1].

### 2.2. Simulation Procedure

ANSYS meshing was employed to perform discretization of the computational domain. Prior to launching CFD simulation, index of mesh skewness was checked to assess meshing quality. In addition, standard mesh convergence studies were performed using the Grid Convergence Index (GCI) method [26]. GCI value was calculated using Equations (1)–(3):

(1) GCI=3|ε|(rp−1)

(2) ε=(f1−f2)f1

(3) r=(NfineNcoarse)1/3

where *ε* is a relative error indicator, *f*_1_ is the variable value calculated using a fine mesh, and *f*_2_ is the variable value at the same point calculated using a coarse mesh. The mesh refinement ratio *r* is calculated in Equation (3), where *N_fine_* and *N_coarse_* are the total number of cells of the fine and coarse mesh, respectively.

Three mesh files of TISE model corresponding to 6.9, 12.1, and 20.7 million cells were generated. Thereby, *r* = 1.2 was the mesh refinement ratio from the middle mesh to the coarse mesh, and *r* = 1.4 was the ratio from the fine mesh to the coarse mesh. The GCI at three selected points P1, P2, P3 were analyzed and compared with the variables of air speed, temperature, and pressure, respectively. The GCI value decreased when r increased from 1.21 to 1.44 (Table 1), indicating the mesh refinement offered improvement [20]. However, the finest mesh demanded excessive computer power, which was not feasible and affordable practically. Therefore, the middle mesh with cell count around 12 million was adopted. The number of total cells for TISE, MICE, MIRE, and MIAE was 12.1 million, 12.4 million, 12.3 million and 12.3 million, and the average skewness was 0.21, 0.21, 0.22, and 0.22, respectively.

To perform steady-state simulations, standard 𝑘 − 𝜀 turbulence model with enhanced wall functions was used for all the simulations. A designated material “layer-body” was created to represent hens with referenced parameters [27]. The air was modeled as an incompressible ideal gas. Numerical solutions were fully converged by 2500 iterations when both the monitoring variable at these selected points and the residual values were stabilized [24].

### 2.3. Post-Processing

**Figure 4:**
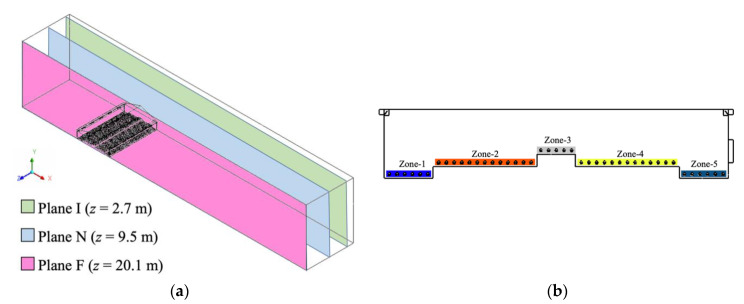
Important locations are shown for data processing and analysis at: (**a**) three designated two-dimensional planes; (**b**) front view of five hen-occupied zones (Plane I, TISE).

Three two-dimensional planes were created to adequately represent all locations in the entire domain (Figure 4a) [1]. These parallel cross-sectional slices along the *z*-axis represented locations impacted by different ventilation features [1,24]. In each plane, a row of bird models was crossed to examine indoor environmental conditions from an bird welfare perspective. In addition, five hen-occupied zones were specified to quantify the environmental parameters (Figure 4). The dimensions of each zone were depicted in the previous publication [1]. Those five hen-occupied zones play an important role in evaluating ventilation scheme performance in ensuring bird welfare, in terms of indoor air conditions and hen comforts.

### 2.4. Bird Welfare Assessment

Simulated indoor air conditions were assessed upon specific comfort criteria from a perspective of bird welfare to evaluate if a certain ventilation scheme could satisfy the requirement. Those requirements [1,3,24] included a desired temperature range between 18 and 24 °C (64 to 75 °F); a range between 0.25 and 1.0 m/s (49 to 197 ft/min) of the air speed at hen level; and a normal static pressure difference of −25 to −10 Pa (−0.10 to −0.04 in. of water) [28] for this type of negative pressure ventilation system.

For cold weather, the desirable range of ventilation rate for the modeled layer house ought to be 0.39 to 1.95 m^3^/s (1404 to 4131 ft^3^/min) [1,24]. Each inlet modeled in this study had an identical opening height [24] to maintain an adequate static pressure difference [28,29].

### 2.5. Statistical Analysis

Predicted environmental data of CFD simulations were quantitatively compared across five hen-occupied zones at every single reference plane. Note that only one data output was exported at a given location since it did not vary with time after convergence for a steady-state analysis. Therefore, exported data points in each zone were treated as repeated measurements captured throughout that hen-occupied zone. The simulation data were fit to a mixed-effects model as shown in Equation (4):

(4) $$y_{ijk}=\mu +\tau_{i}+\beta_{j}+{\left(\tau \beta \right)}_{ij}+\varepsilon_{ijk}$$

$$i=1,\;\ldots ,\;a_{E};j=1,\;\ldots ,\;b_{E};k=1,\;\ldots ,\;c_{E}$$

$$\varepsilon_{ijk}\;i.i.d.\;N\left(0,\;\sigma_{1}^{2}\right)$$

To compare the simulation data between models, the primary interest was testing whether different models had different results at the same plane. Thus, the interpretation for Equation (4) is: μ was the mean of a parameter (air speed, temperature, pressure), $\tau_{i}$ was the effect of the 𝑖th model, $\beta_{j}\;$ was the effect of the 𝑗th zone, ${\left(\tau \beta \right)}_{ij}\;$ was the interactive effect of model and zone. Herein, $\varepsilon_{ijk}$ was the random error in terms of independent and identically distributed (i.i.d.) random variables. In addition, 𝐸 took a value in {1,2,3} and referred to the number of a particular plane. The values $a_{E}$ and $b_{E}$ were 4 and 5, representing the number of levels of factors: model and zone. The value of $c_{E}$ referred to the number of levels of the data points, varying over the three planes. Furthermore, the null hypotheses were:

- ***H_0_*** *: there was no difference in the means of factor Model.*
- ***H_0_****: there was no difference in the means of factor Zone.*
- ***H_0_****: there was no interaction between factors Model and Zone.*

The hypotheses were tested using analysis of variance (ANOVA) using R Studio v1.2 [30]. A *p*-value significance level α = 0.05 was used for determining whether $H_{0}$ would be rejected. In addition, pairwise analysis between means of interest were conducted with Tukey’s multiple comparison procedure [31].

## 3. Results

Four ventilation schemes were analyzed and compared with contours of airflow patterns, temperature distribution, and static pressure difference. In addition, environmental data of five different zones at hen level were statistically analyzed to compare and assess the bird welfare suitability of indoor air conditions provided by each design.

### 3.1. Airflow Analysis

**Figure 5:**
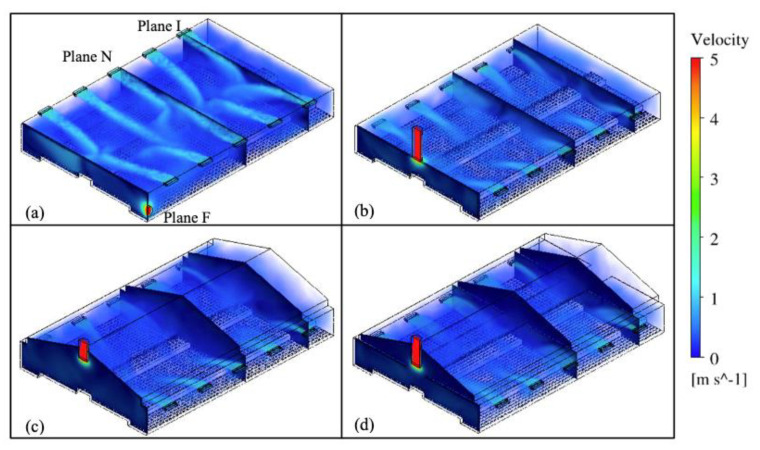
Isometric view of the study layer house showing the location of three reference planes as indicated in (1), and overall airflow patterns and air velocity magnitudes within four ventilation schemes: (**a**) TISE, (**b**) MICE, (**c**) MIRE, (**d**) MIAE.

**Figure 6:**
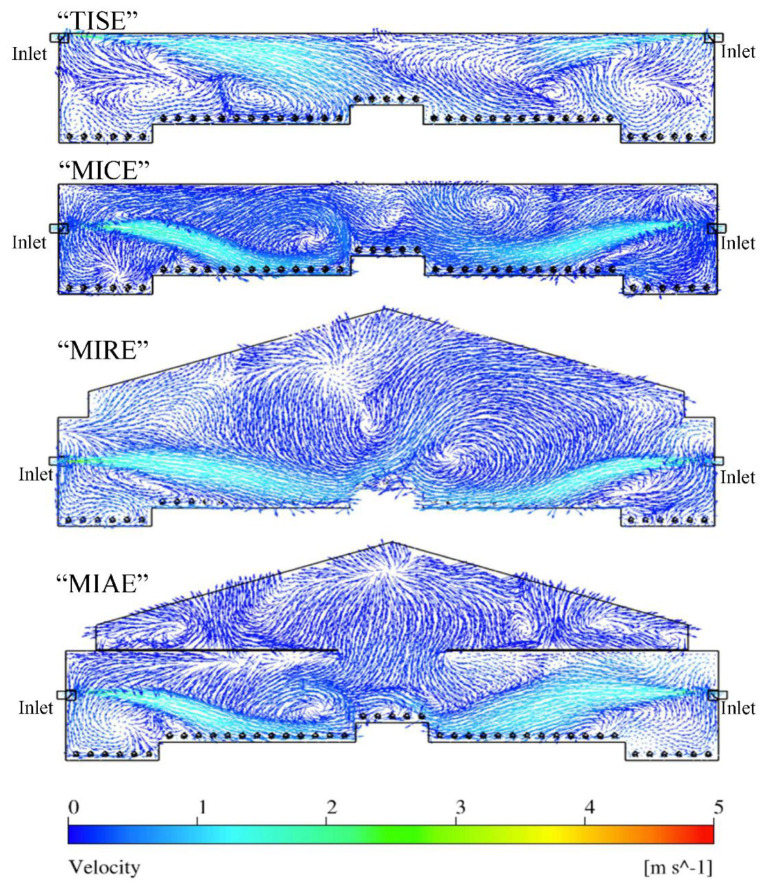
Indoor air velocity vector contours at Plane I of four models.

Three-dimensional rendering of air velocity magnitude of the entire layer house and velocity vectors at each reference plane were created to visualize indoor airflow patterns (Figure 5). In general, fast incoming air jets were observed from both inlets in all four ventilation schemes, and gradually decreased their magnitudes approaching the nest-boxes area. Although incoming air jets of the upwind inlet showed longer trajectories compared to those of downwind inlets, obvious air circulations were observed throughout the house within four schemes (Figure 6).

The patterns of indoor airflow varied with the type of ventilation schemes at Plane I. The standard TISE ventilation system possessed the strongest incoming air jets compared to three alternative designs, because the TISE model had inlets at the top of the sidewalls along a flat ceiling (Figure 5 and Figure 6). Without the ceiling along which the air jets could move, the fresh air trajectories in the other three models more quickly dropped towards the hen occupied area after traveling fairly horizontal paths for a short distance. Another observation was that the incoming air from the downwind (right-side) inlet had relatively smaller size except for the MIAE model (Figure 6). In addition, strong and numerous air circulations were observed throughout the house in all models. For the three alternative designs, circulations accumulated particularly at the central area, while the majority of air circulations concentrated at areas close to both sidewalls in TISE model. In contrast, hen-occupied area over the slatted floor, besides nest-boxes in MICE, MIRE, and MIAE had fast moving airflows derived from incoming air jets (Figure 6).

**Figure 7:**
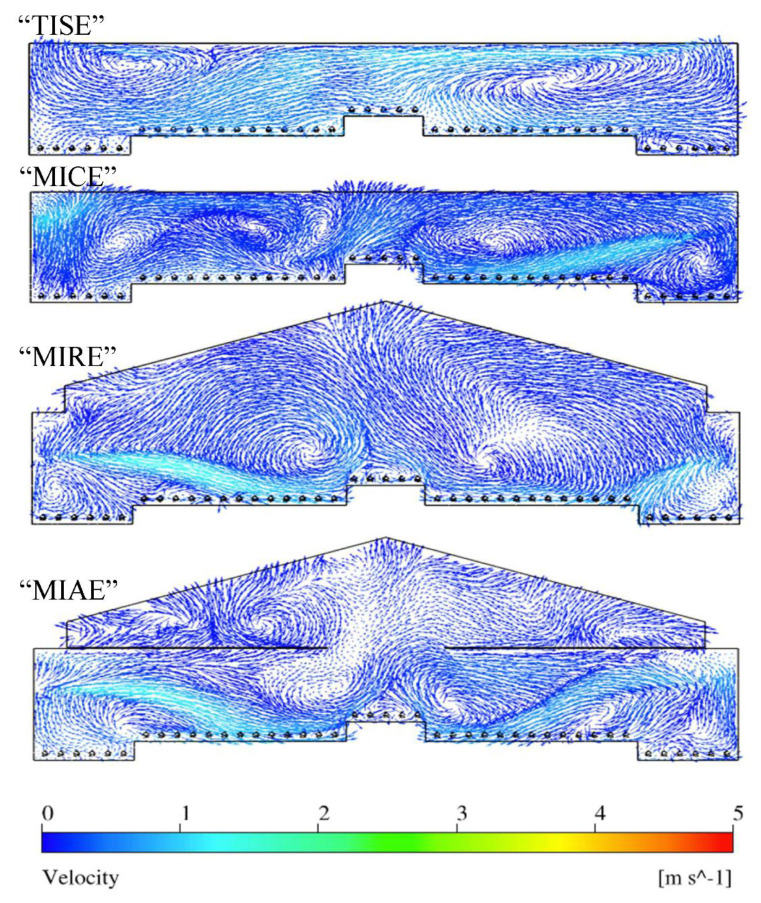
Indoor air velocity vector contours at Plane N of four models.

Air movement patterns varied dramatically at Plane N that contained no ventilation features (Figure 7). Vigorous airflows moved from the upper right to the lower left of the house in TISE [1]. In the MICE model, strong air movements were observed at the right portion of the house close to the sidewall, moving towards the central nest-box area. There was also a small portion of fast airflow at the upper left in MICE, which is close to an obvious swirl nearby. The MIRE model had strong airflows starting from the left portion of the house, where the position and the trajectory of the airflows were coincident to the upwind incoming air jet. Even vigorous airflows with similar trajectories were observed in the MIAE model from both sides. Sufficient internal airflow mixing and the impact from incoming fresh air nearby resulted in an overall suitable air movement at Plane N. Because Plane N represented the majority (69%) [1] of cross-sectional locations in the layer house, air movements in this Plane would be more representative of overall house conditions.

**Figure 8:**
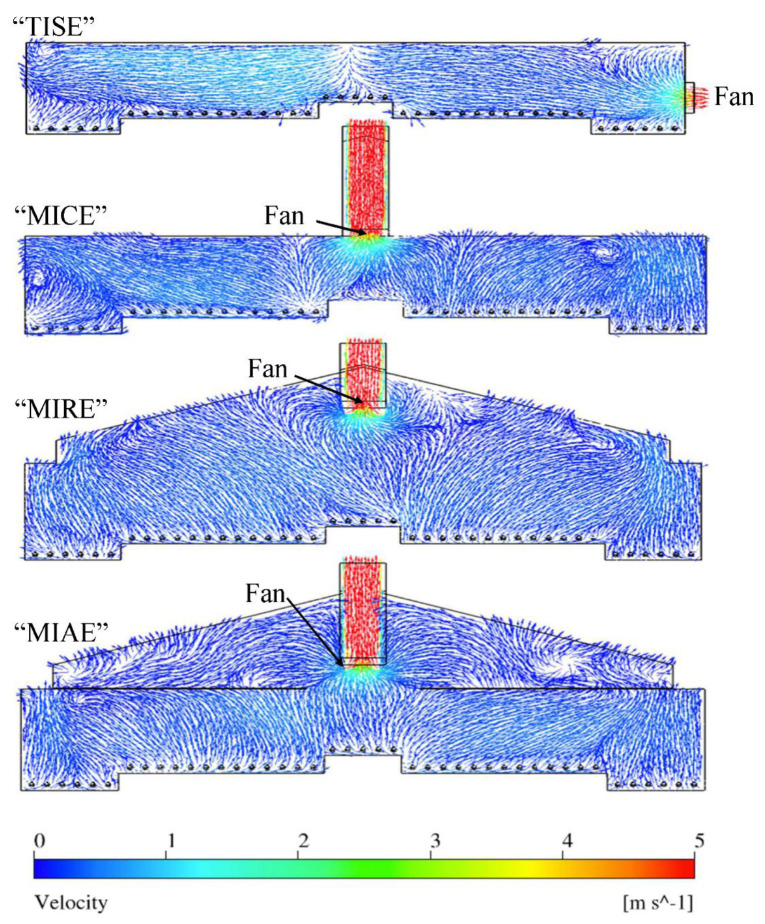
Indoor air velocity vector contours at Plane F of four models.

The air movement patterns at Plane F exhibited the influence of the exhaust fan on performance of each model (Figure 8). The TISE model presented different patterns compared to the other three models due to the distinct sidewall location of the fan. Air speeds at the right portion of the layer house increased gradually as approaching the fan area in TISE. However, a vigorous horizontal airflow was observed within the left portion of the house with even higher magnitudes. For models MICE, MIRE, and MIAE, uniform patterns of air movement were found towards the upper portion of the house because of the driving force from the exhaust fan. However, some slanted airflows were observed in the MICE model moving from the nest-box area to the upper left corner. In the MIAE model, most of the attic space was filled with airflows moving upwards, excluding some airflows close to the duct and fan areas. Unlike the air circulations that formed close to the left sidewall in the TISE model, strong air circulations were observed largely in the MICE model and the attic area of MIAE. Simulations confirm the importance of relative locations of inlets and exhaust fans in determining overall indoor airflow patterns. However, note the limited influence of a fan beyond a few meters (fan diameters) into the hen house.

### 3.2. Temperature Analysis

**Figure 9:**
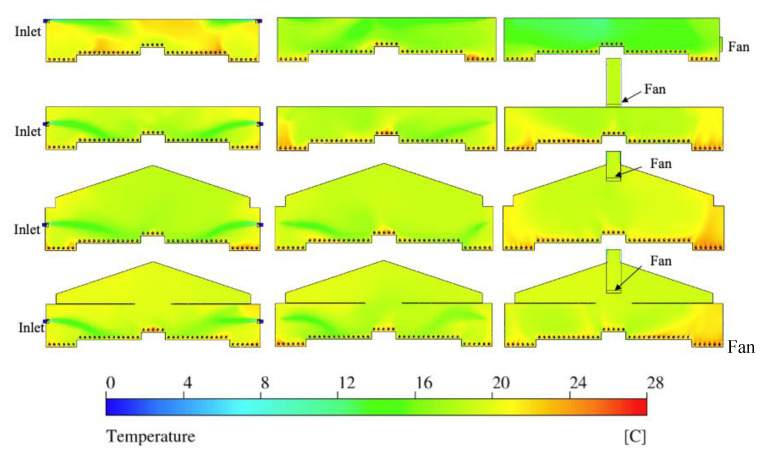
Indoor temperature contours of four models, from top to bottom rows: TISE, MISE, MICE, and MIAE at three reference planes (columns from left to right: Plane I, Plane N, and Plane F).

Although no artificial heating was supplied in the layer house in a cold weather, indoor temperature was maintained warm due to birds’ body heat. The distribution of temperature at each plane was analyzed by temperature contours (Figure 9).

The incoming fresh air (dark blue) with an initial temperature of 0 °C (32 °F) at both inlets ran into a quick color transition after mixing with indoor air, approaching the nest-boxes area (Figure 9). At Plane I, larger regions of orange and yellow warmer temperatures were observed in the TISE model, particularly at the hen level and the center upper portion of the layer house. An explanation is that buoyant currents lifted warm air from ground, which was then not adequately mixing with the incoming cold air. However, for all three alternative ventilation systems, the incoming cooler air was introduced quite directly to the hen-occupied slatted floor area at both sides of the nest-boxes (Figure 9). Some portions at the hen level in three alternative models were observed at temperatures (approximately 14 to 16 °C; 57.2 to 60.8 °F) lower than desirable range, yet warm air accumulated at the litter area close to both sidewalls. Unlike MICE and MIRE, relative warmer portions were at the upper right corner and nest-boxes area in the MIAE model.

The reason for the different patterns between the TISE model and the other three models was the position of inlets. The inlet at a lower, mid-wall position in the house of MICE, MIRE, and MIAE provided cold air that was quickly mixed with the warm air that was heated by the birds. Hence, the warmed air did not have a chance to rise toward upper areas of the interior due to mixing into the cooler inlet air. Otherwise, the incomplete mixing of cold incoming air and warm house air in TISE left warmer air stratified near the ceiling location of low airflow. This explained why the obvious warm region was not observed in the other models.

The indoor temperature distribution at Plane N (Figure 9) contained larger green (12 to 16 °C; 53.6 to 60.8 °F) area in TISE at the upper portion and the hen level on the left side, compared to the other models. The three alternative designs tended to have similar patterns that were found at Plane I (Figure 8), demonstrating the incoming cold air from adjacent inlets had solid impacts on indoor temperature distribution. Most warm regions were observed at the hen level, although of varied locations in each model. For instance, the largest warm area in TISE was located close to the right sidewall, while the MICE and MIAE models had their warmest region close to the left sidewall and nest-boxes. The distinguishable warmest area in MIRE was found at the center of nest-boxes region. However, one common issue at Plane N might be the undesired low temperature regions at some bird-occupied areas in each model (Figure 9).

The temperature contours of TISE at Plane F showed large areas of relatively low temperature (10 to 16 °C; 50 to 60.8 °F) with the majority of the house appearing green on the color-temperature scale (Figure 9). The three alternative designs had larger areas with warm temperature compared to TISE, particularly at the hen-occupied region at both sides of the layer house rather than central areas. Moreover, clear temperature gradients can be seen in contours of MICE, MIRE, and MIAE at both sides. The fan zone and the duct space had temperatures about 18 to 20°C (64.4 to 68 °F), and the distribution was quite uniform as illustrated. In general, the uniformity of indoor temperature of the TISE model was better than the other three models. However, the temperature on average was warmer at the hen level within three alternative ventilation schemes. Quantitative analysis was conducted to interpret these differences (Section 3.4).

### 3.3. Pressure Analysis

**Figure 10:**
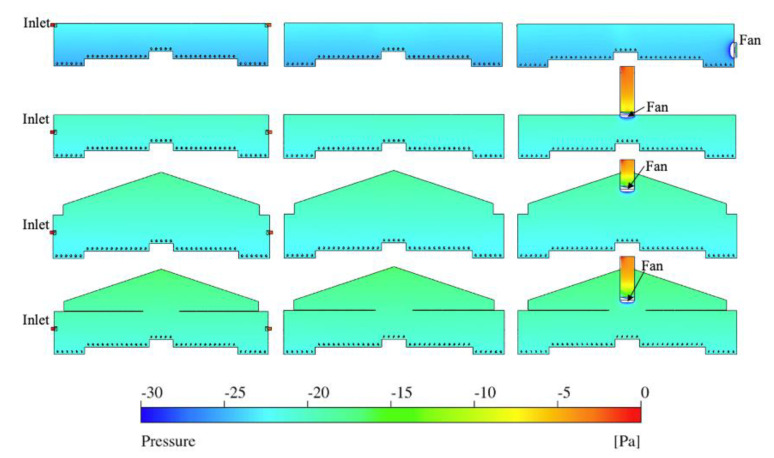
Indoor pressure contours of four models, from top to bottom rows: TISE, MICE, MIRE, and MIAE at three reference planes (columns from left to right: Plane I, Plane N, and Plane F).

Static pressure difference between interior and exterior of the study layer house varied slightly over four ventilation schemes with a total range from −30 to 0 Pa (the atmospheric pressure was set at 0 Pa) (Figure 10). The uniformity of static pressure was acceptable for individual models, while slight differences were observed between distinguishable ventilation systems. Overall, the static pressure was very uniform within each ventilation scheme.

Indoor static pressure in the TISE model was −24.3 Pa at Plane I on average. The redness at both inlets indicated the attachment to outdoor atmosphere. The pressure of MICE and MIRE fell in the range of −20 to −24 Pa, while the indoor static pressure of MIAE had the smallest magnitude, ranging between 18 to 22 Pa. Overall average static pressure fell in a normal range [29]. Identical ranges and uniformity of indoor static pressure were also observed from the contours at Plane N within all four ventilation schemes (Figure 10).

In addition, the indoor static pressure at Plane F showed similar patterns compared to the other two planes excluding the white region close to the exhaust fan where the static pressure lower than −30 Pa (−56 Pa, −59 Pa, −75 Pa, −73 Pa for TISE, MICE, MIRE, and MIAE, respectively).

### 3.4. Hen Comfort Assessment

Environmental conditions in terms of air speed, temperature, and static pressure at the hen level were analyzed quantitatively to evaluate the performance of each ventilation scheme in ensuring hen comfort. Simulation outputs were analyzed at each hen-occupied zone separately. Because all these data varied by indoor locations, referring to the type of planes, comparisons across four models ought to be conducted at the same plane. Thereby, statistical analyses were performed to test the significance of key factors’ effects on the simulation data, including the type of model, the zone, and their interactive effects.

At Plane I, in total, 35,340 data points were exported for thorough analysis from four models’ simulation results. The ANOVA analysis suggested that for all environment parameters, the effects of model type, hen-occupied zone, and the interactions among them, were statistically significant (Table 2) according to the F-value and *p*-value, accordingly.

Same ANOVA procedure was conducted to analyze the data at Plane N and Plane F exported from simulation results of each model (Table 3 and Table 4). The data set size varied slightly as 34,986 and 34,178 data points from Plane N and Plane F, respectively. Results of ANOVA analyses in Table 3 and Table 4 demonstrated the effects of model type and zone, and their interactions were statistically significant. All these analyses showed that the ventilation schemes and the hen-occupied area were both crucial to the indoor environment.

**Figure 11:**
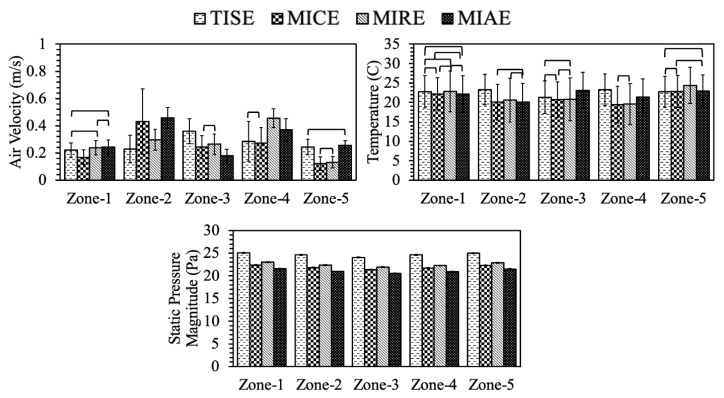
Environmental parameters of hen-occupied zones at **Plane I** in four models. Crossbars indicate **No** statistically significant differences at 95% family-wise confidence level. Error bars stand for the standard deviation.

**Figure 12:**
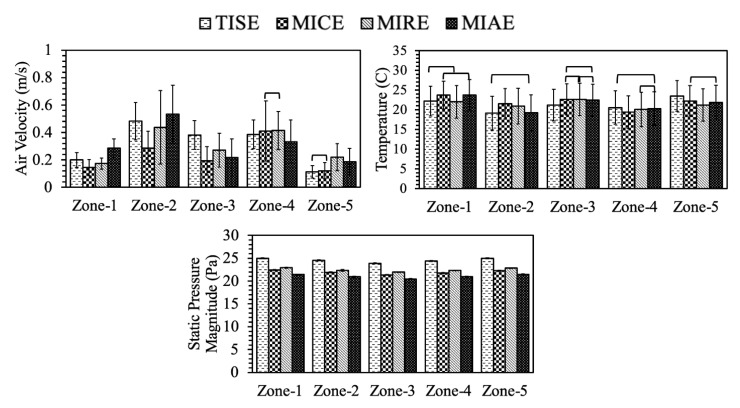
Environmental parameters of hen-occupied zones at **Plane N** in four models. Crossbars indicate **no** statistically significant differences at 95% family-wise confidence level. Error bars stand for the standard deviation.

**Figure 13:**
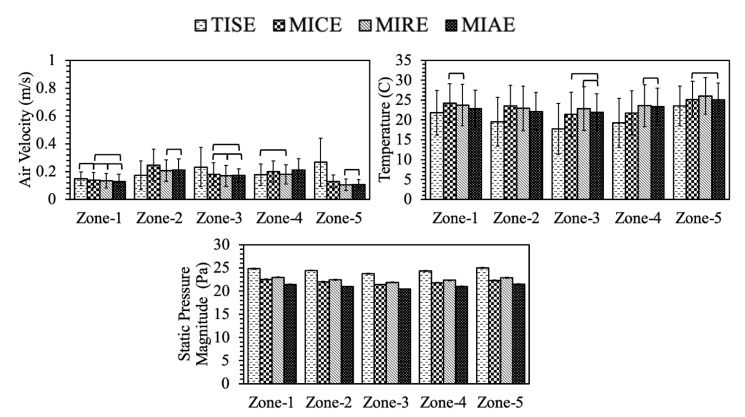
Environmental parameters of hen-occupied zones at **Plane F** in four models. Crossbars indicate **no** statistically significant differences at 95% family-wise confidence level. Error bars stand for the standard deviation.

As follow-up analyses, Tukey’s tests were applied in pairwise comparisons for parameters of interest. The results of Tukey’s test were reflected in Table 5, Table 6 and Table 7 and graphed in Figure 11, Figure 12 and Figure 13 by comparing the average value of each parameter between individual models at the same plane.

At Plane I, MIAE had the fastest average air speed in hen-occupied area compared to the other models (Table 5), and the slowest average air speed was observed in TISE. Relatively swift air speeds were observed in the middle of the house for all models, referring to zones 2, 3, and 4 as illustrated in Figure 12. This might be explained as the incoming air jets likely reached these zones after mixing with house air circulations (Figure 6). Note, gentle airflow at 0.25 m/s (50 fpm) was observed at hen level and was desired during cold weather as adequate air movement was provided without chilling the hens. At Zone-1 and Zone-5, no significant difference was observed between the standard design and three alternatives. However, TISE had the fastest air speeds at the center next-box Zone-3, compared to the alternatives. At Zone-2 and Zone-3, MIAE and MIRE possessed the maximum average air speeds, respectively. Generally, these results were consistent with the paths of air jets in Figure 5.

Average temperature of TISE was the warmest at Plane I, about 22.90°C at the hen level, which was 2.26 °C higher than the lowest of MICE. Interestingly, the average temperature of MIAE was the second highest, though it had the fastest air speed on average. Average temperature at each zone was exceptionally close between four models as several crossbars indicating the lack of statistical significance at Plane I (Figure 11). For instance, at Zone-1, no pairwise difference was statistically significant. Air temperature at hen-occupied areas with the standard ventilation scheme (TISE model) tended to be slightly warmer than the other three models at Zone-2 and Zone-4, although the average temperature within MIAE model was the highest at Zone-3 and Zone-5. These analyses showed consistent results with Figure 8, as yellowish and reddish warm regions were fairly obvious in corresponding positions.

Indoor static pressure averages at hen level were of extraordinary uniformity within each zone for individual model as small standard deviations suggested (Figure 11). Moreover, the magnitudes from different models presented exceptional consistency with a range of only 3.62 Pa (Table 5). Indoor pressure of the standard TISE configuration possessed the maximum magnitude with statistical significance. All four models showed pressure ranges per the evaluation criteria [28,29].

Simulation data from Plane N revealed different patterns at hen-occupied zones (Table 6). Interestingly, the average air speeds slightly increased except for MICE, compared to those of Plane I. One overall pattern was relatively higher air speeds at Zone-2 and -4 for all the models, referring to the regions beside the center nest-boxes, where vigorous airflows existed (Figure 7). Moreover, the difference between TISE and MIAE had no statistical significance, nor did the velocity between TISE and MIRE. As Table 6 suggests, the average air speed was 0.27 m/s within the MICE ventilation scheme, which was the lowest at hen level among the four models. In general, more still air was observed at Plane N, which was not surprising since no ventilation features existed at this plane. Particularly, the air speeds within MICE configuration were relatively slower than the others at four zones expect for Zone-4, which was reasonable as weaker air movements were observed at those regions as Figure 7 suggested. Another overall pattern was relatively higher air speeds at Zone-2 and -4 for all the models, referring to the regions aside the center nest-boxes, where vigorous airflows existed (Figure 7). Recalling Plane N represented 69% cross-sections of the entire poultry house, the air speeds from this plane were actually higher than those of Plane I, which means sufficient air mixing can result in robust air movements at hen level even without direct incoming air.

The average temperatures of each model were fairly uniform at Plane N. The highest mean temperature was in the MICE model as it had the slowest air speed on average (Table 6). Additionally, no statistical significances were found between temperature means of TISE and MIAE, nor between MIRE and MIAE. The trend of temperature distribution among five zones showed the reverse trend to that of air speed in general. Average temperature at Zone-1 and Zone-5 were slightly higher than those of the other zones, which worked for all models. At Zone-3, three alternative models had extremely close mean with no statistical significance. At symmetrical Zone-2 and -4, the average temperature within standard TISE configuration showed no statistically significant difference with that of MIAE. In addition, the warmest temperature on average at Zone-4 and Zone-5 were both found within TISE, while the coolest was observed within MICE and MIRE, respectively. As Figure 9 depicts, almost no warm yellowish or reddish regions can be observed at Zone-2 for TISE, resulting in a clear lower temperature on average (Figure 12).

The static pressure at Plane N was identical to the performance at Plane I for each model. The lowest pressure magnitude on average was 21.05 Pa from the data of MIAE, which was the same with that of Plane I. Additionally, the highest magnitude was 24.53 Pa of TISE model.

Simulation results at Plane F suggested all four models have fairly uniform air speeds with relatively smaller magnitudes (Table 7). All four models had statistically identical air speeds at Zone-1. At Zone-3, the highest mean of air speeds was observed in the standard TISE mode, while the other three models had statistically identical results (Figure 13). Similarly, the TISE model had the highest average air speed at Zone-5 with large variation, which reflected the drastic influence from the exhaust fan nearby. In addition, TISE showed the slowest average air speeds at Zone-2 and -4 among the four models due to a lack of robust airflows as illustrated in Figure 7. Although the exhaust fan would have a strong impact on airflows at this plane, the air speeds at hen level turned out to be considered still air.

Average temperature at Plane F increased slightly due to slower air speeds overall. The temperature of TISE was the lowest on average about 20.10 °C, while MIRE had the highest temperature of 23.65 °C, which was consistent with its low average air speeds (Table 7). The differences between five hen-occupied zones were quite small. Temperatures tended to decline at the middle of Plane F, so the average temperature at Zone-3 was the lowest of each model. In addition, the temperature at hen level within the standard TISE model was lower than the alternative models at all five zones with statistical significance as depicted in Figure 13. 

Indoor static pressure at hen level at Plane F was identical to the other two planes (Table 7) and was quite uniform within each ventilation scheme with rather small variations at different zones (Figure 13).

## 4. Discussion

Three alternative ventilation schemes presented comparable performances with the standard TISE, in terms of sufficient indoor airflow and air mixing. By observing air velocity vector contours, MICE, MIRE, and MIAE were able to provide strong incoming air jets that can reach the central region of the house almost as well as the TISE model. Simulation results of the four ventilation schemes revealed a total ventilation rate of 1.97 m^3^/s (4174 ft^3^/min), 1.93 m^3^/s (4089 ft^3^/min), 1.96 m^3^/s (4153 ft^3^/min), and 1.91 m^3^/s (4047 ft^3^/min) for the study layer house respectively, which were on the high end of the recommended range (0.39 to 1.95 m^3^/s). Furthermore, the alternative designs provided indoor air movements similar to the standard model, even better at the hen level (Plane I). Air speeds on average were maintained at 0.35 m/s (69 ft/min) at hen level in the majority of the house for TISE, MIRE, and MIAE. Airflow pattern visualization showed two large circular air eddies that included the incoming air jets in TISE whereas the alternative models had these large eddies along with more numerous, smaller circulation patterns.

Temperature contours of individual models indicated that the uniformity of indoor temperature distribution was satisfactory overall. The entire indoor temperature was kept above 15 °C on average, assuming the house was ideally insulated. Nonetheless, all four models presented the capacity to ensure birds comfortable temperatures. The average temperature at the hen level varied within a range of approximately 21 to 24 °C for the three alternative models, which matched the comfort zone for birds nearly perfectly. Furthermore, the average temperature at hen-occupied areas of the three alternative models was slightly higher than that of the TISE model at reference planes N and F. In particular, within Zone-3 (where nest-boxes were located) should be of interest, as the TISE model offered higher air speed and lower temperature than the alternative designs. Microenvironment in and around the nest-boxes may be used to encourage nest-box use by hens rather than laying eggs in other parts of the house.

The static pressure of the house was quite uniform for each model and within a normal range. Slight differences among models up to 4 Pa were found. The TISE model had the largest magnitude of static pressure around 25 Pa, while the MIAE model had the smallest magnitude of static pressure. In addition, static pressure at different locations for the same model was quite consistent.

Future studies may be conducted with regard to design details of cage-free hen housing accordingly. As revealed in this work, airflow patterns and the formation of indoor air circulations were highly dependent on the trajectory of incoming air. The relative location of inlets and exhaust fans, the dimensions of baffles, and so on, all play an indispensable role in the formation of indoor environment. In addition, further CFD analysis can be used to examine the uniformity of temperature and air speeds as well as the environmental parameters at the hen level to reason and address practical problems, such as floor eggs.

## 5. Conclusions

One standard and three alternative ventilation schemes were modeled at full-scale to simulate indoor environmental conditions in a commercial floor-raised cage-free hen house with the goal of evaluating the performance of ventilation scheme in maintaining comfortable conditions for good bird welfare.

CFD simulation results suggested three alternative ventilation schemes have competitive performance compared to the standard scheme, and demonstrated impacts of the ventilation schemes on characteristics and uniformity of indoor environmental conditions. Data of environmental parameters at the hen level were documented to ensure hen comfort. The performance of alternative ventilation systems was assessed accordingly. Planes I and N had the most variability of air speeds due to the impact of fresh air inlet in or adjacent. The middle zones of each house (Zone-2, -3, and -4) in Planes I and N had the highest air velocities and largest variation in air velocities, yet they were below the threshold for being considered chilling drafts on the birds. Higher air speed in this central area was a result of cooler, fresh incoming air circulating into that portion of the layer house. However, fortunately, there was not a trend toward cooler temperatures within those bird-occupied zones at the middle of the house, which indicated the central nest-boxes area would be favored for laying eggs. Temperatures within the animal zones were variable across all zones (high standard deviations), but on average showed reasonably uniform temperatures within each model and across models. Static pressure had the smallest variation among planes and zones within a model. The static pressure data suggested consistency at each zone of an individual model, implying a clear trend of the average magnitude of pressure from high to low: TISE, MICE, MIRE, and MIAE, ranging from only 21 to 25 Pa.

This study recognizes CFD modeling is a robust methodology to analyze ventilation performance and assess bird welfare conditions. Full-scale modeling with individual simplified animal models enforces the usefulness of CFD simulation and restores the model’s realism. The four models herein can be fine-tuned to assess other existing ventilation schemes or evaluate proposed ventilation options for various types of poultry houses. In summary, CFD modeling facilitates investigators to tackle animal welfare problems and explore sophisticated solutions related to animal housing.

## Figures and Tables

**Table 1:** GCI calculated for common values of mesh refinement ratio ( r ) with second order upwind scheme (TISE).

Points	Location			GCI	
	x	y	z	r=1.44	r=1.20
P1	252	105	108	121.75%	224.07%
P2	0	50	373	1.99%	4.06%
P3	0	85	500	5.02%	6.73%

**Table 2:** ANOVA of environmental data from five hen-occupied zones at Plane I.

Parameter	Factor	Degree of Freedom	Sum of Squares	Mean Square	F-Value	Pr (>F)
Air speed	Model	3	32	11	403	<2.2 × 10^−16^
	Zone	4	155	39	1474	<2.2 × 10^−16^
	Model × Zone	12	161	13	510	<2.2 × 10^−16^
	Residuals	35,320	928	0.03		
Temperature	Model	3	24,506	8169	406	<2.2 × 10^−16^
	Zone	4	25,069	6267	311	<2.2 × 10^−16^
	Model × Zone	12	26,273	2189	109	<2.2 × 10^−16^
	Residuals	35,320	711,278	20		
Pressure	Model	3	64,188	21,396	1,452,264	<2.2 × 10^−16^
	Zone	4	3419	855	58,018	<2.2 × 10^-16^
	Model × Zone	12	69	6	390	<2.2 × 10^−16^
	Residuals	35,320	520	0		

**Table 3:** ANOVA of environmental data from five animal-zones at Plane N.

Parameter	Factor	Degree of Freedom	Sum of Squares	Mean Square	F-Value	Pr (>F)
Air speed	Model	3	41	14	622	<2.2 × 10^−16^
	Zone	4	389	97	4408	<2.2 × 10^−16^
	Model × Zone	12	105	9	399	<2.2 × 10^−16^
	Residuals	34,966	771	0.02		
Temperature	Model	3	1637	546	31	<2.2 × 10^−16^
	Zone	4	47,323	11,831	682	<2.2 × 10^−16^
	Model × Zone	12	19,390	1616	93	<2.2 × 10^−16^
	Residuals	34,966	606,596	17		
Pressure	Model	3	57,891	19,297	142,316	<2.2 × 10^−16^
	Zone	4	3418	855	63,049	<2.2 × 10^−16^
	Model × Zone	12	45	4	278	<2.2 × 10^−16^
	Residuals	34,966	474	0		

**Table 4:** ANOVA of environmental data from five animal-zones at Plane F.

Parameter	Factor	Degree of Freedom	Sum of Squares	Mean Square	F-Value	Pr (>F)
Air speed	Model	3	3	1	141	<2.2 × 10^−16^
	Zone	4	27	6	797	<2.2 × 10^−16^
	Model × Zone	12	30	3	353	<2.2 × 10^−16^
	Residuals	34,158	243	0.007		
Temperature	Model	3	64,525	21,508	767	<2.2 × 10^−16^
	Zone	4	44,765	11,191	399	<2.2 × 10^−16^
	Model × Zone	12	12,750	1063	38	<2.2 × 10^−16^
	Residuals	34,158	957,499	28		
Pressure	Model	3	53,896	17,965	1,701,164	<2.2 × 10^−16^
	Zone	4	3573	893	84,583	<2.2 × 10^−16^
	Model × Zone	12	83	7	656	<2.2 × 10^−16^
	Residuals	34,158	361	0		

**Table 5:** Means of environment parameters at **Plane I** in four models. Note all the differences are statistically significant (ANOVA and subsequent Tukey’s test, *p* < 0.01) in this table.

Model	Number of Data Points	Air Speed (m/s)	Temperature (°C)	Static Pressure Magnitude (Pa)
TISE	8747	0.26	22.90	24.67
MICE	8737	0.28	20.64	21.87
MIRE	8578	0.31	21.16	22.45
MIAE	9278	0.34	21.53	21.05

**Table 6:** Means of environment parameters at **Plane N** in four models. Note all the differences without annotation are statistically significant (ANOVA and subsequent Tukey’s test, *p* < 0.01) in this table.

Model	Number of Data Points	Air Speed (m/s)	Temperature (°C)	Static Pressure Magnitude (Pa)
TISE	8630	0.35 ab	20.84 c	24.53
MICE	8646	0.27	21.4	21.91
MIRE	8579	0.34 a	21.04 d	22.43
MIAE	9131	0.35 b	20.91 cd	21.05

**Table 7:** Means of environment parameters at **Plane F** in four models. Note all the differences without annotation are statistically significant (ANOVA and subsequent Tukey’s test, *p* < 0.01) in this table.

Model	Number of Data Points	Air Speed (m/s)	Temperature (°C)	Static Pressure Magnitude (Pa)
TISE	8494	0.19 a	20.10	24.45
MICE	8439	0.19 a	23.05 b	21.96
MIRE	8440	0.17	23.65	22.47
MIAE	8805	0.18	22.97 b	21.02
