# Correction: Chen et al. Computational Fluid Dynamics Analysis of Alternative Ventilation Schemes in Cage-Free Poultry Housing. *Animals* 2021, *11*, 2352

**DOI:** 10.3390/ani12172175

**Published:** 2022-08-25

**Authors:** Long Chen, Eileen E. Fabian-Wheeler, John M. Cimbala, Dan Hofstetter, Paul Patterson

**Affiliations:** 1 Department of Agricultural and Biological Engineering, The Pennsylvania State University, State College, PA 16802, USA; 2 Tianjin Academy of Agricultural Sciences, Tianjin 300384, China; 3 Department of Mechanical Engineering, The Pennsylvania State University, State College, PA 16802, USA; 4 Department of Animal Science, The Pennsylvania State University, State College, PA 16802, USA

## Text Correction

There was an error in the original publication [1]. A correction was needed for the last sentence in Section 2.1.5. “Boundary Conditions” on Page 6:

“A constant pressure jump of 18 Pa (0.072 in. of water) was applied across all the cells in the fan zone [1].”

This is incorrect because each of the four models has a unique value of pressure jump. The sentence was cut off by mistake and not caught during proof-reading for some reason.

A correction has been made to this sentence in Section 2.1.5 on Page 6:

“Constant pressure jump values of 18 Pa (0.072 in. of water), 20 Pa (0.080 in. of water), 16 Pa (0.064 in. of water), and 15 Pa (0.060 in. of water) were applied across all the cells in the fan zone of TISE, MICE, MIRE, and MIAE models, respectively [1].”

The authors apologize for any inconvenience caused and state that the scientific conclusions are unaffected. This correction was approved by the Academic Editor. The original publication has also been updated.
